# Abernethy malformation associated with Caroli’s syndrome in a patient with a *PKHD1* mutation: a case report

**DOI:** 10.1186/s13000-017-0647-y

**Published:** 2017-08-16

**Authors:** Xiao-xiao Mi, Xiao-guang Li, Zi-rong Wang, Ling Lin, Chun-hai Xu, Jun-ping Shi

**Affiliations:** 1 Institute of Translational Medicine, the Affiliated Hospital of Hangzhou Normal University, Hangzhou, Zhejiang China; 2 Department of Infectious Diseases, The Second Affiliated Hospital of Harbin Medical University, Harbin, Heilongjiang China; 3 Department of Radiology, The Affiliated Hospital of Hangzhou Normal University, Hangzhou, Zhejiang China; 4 Shanghai Biotecan Medical Diagnostics Corporation, Shanghai, China

**Keywords:** Abernethy malformation, Caroli’s syndrome, Liver biopsy, *PKHD1*

## Abstract

**Background:**

Abernethy malformation is a rare congenital anomaly characterised by the partial or complete absence of the portal vein and the subsequent development of an extrahepatic portosystemic shunt. Caroli’s disease is a rare congenital condition characterised by non-obstructive saccular intrahepatic bile duct dilation. Caroli’s disease combined with congenital hepatic fibrosis and/or renal cystic disease is referred to - Caroli’s syndrome. The combination of Abernethy malformation and Caroli’s syndrome has not been reported previously.

**Case presentation:**

We present the case of a 23-year-old female who was found to have both type II Abernethy malformation and Caroli’s syndrome. Radiological imaging was performed, including computed tomography with three-dimensional reconstruction and magnetic resonance imaging with (magnetic resonance cholangiopancreatography (MRCP), which revealed a side-to-side portocaval shunt, intrahepatic bile duct dilation, congenital hepatic fibrosis, and renal cysts. In addition, *PKHD1* (polycystic kidney and hepatic disease 1) gene mutational analysis revealed a paternally inherited heterozygous missense mutation (c.1877A > G, p.Lys626Arg). A liver biopsy confirmed the pathological features of Caroli’s syndrome.

**Conclusions:**

To our knowledge, this is the first reported case of a patient with both type II Abernethy malformation and Caroli’s syndrome diagnosed using a comprehensive approach that included imaging, mutational analysis, and liver biopsy. Additionally, this is the second reported case to date of an Asian patient presenting with liver and renal disorders with the same paternally inherited *PKHD1* missense mutation.

This case has been presented in abstract form at the 26th Annual Conference of APASL, 2017 [1].

## Background

Abernethy malformation is an extremely rare congenital malformation characterised by an extrahepatic portosystemic shunt. It was first described by John Abernethy in 1793 [2]. Since then, fewer than 200 cases have been reported, and the majority of affected patients were <18 years of age and female [3]. Adult patients with this malformation experience various symptoms, including nausea, vomiting, fatigue, epigastric pain, anorexia, jaundice, and dyspnoea. Abernethy malformation can be classified into two types based on the presence of the portal vein and its anastomosis with the inferior vena cava (IVC). Type I is characterised by the congenital absence of the portal vein and a complete end-to-side portocaval shunt; this type is predominantly found in females [4, 5]. Type II is characterised by the presence of a hypoplastic portal vein, which leads to liver perfusion via a partial portal venous supply and a partial side-to-side shunt [6]. Type I is further classified into type IA and type IB, which are defined as malformations in which the superior mesenteric and splenic veins drain separately into the inferior caval vein (type IA) or drain from a common trunk (type IB) [4]. Abernethy malformation, especially type I, is frequently associated with other congenital anomalies, including abnormalities in the cardiovascular system, liver, brain, skeleton, and urogenital tract [7–9]. No cases of type II Abernethy malformation associated with Caroli’s syndrome (a combination of Caroli’s disease and congenital hepatic fibrosis and/or kidney cysts) have been previously reported. Caroli’s disease is a rare congenital disorder characterised by non-obstructive saccular intrahepatic bile duct dilation, and it has a prevalence of 1:1,000,000 in the general population [10, 11]. It is well-known that congenital hepatic fibrosis and/or polycystic kidney disease are closely related to *PKHD1* gene mutations [12, 13].

Here, we present the case of a 23-year-old female with type II Abernethy malformation and Caroli’s syndrome, which were identified after a workup that included medical imaging (computed tomography (CT) and magnetic resonance imaging (MRI)), a liver biopsy and genetic testing. The association of Caroli’s syndrome with Abernethy malformation has not been previously reported in the literature.

## Case presentation

**Fig. 1:**
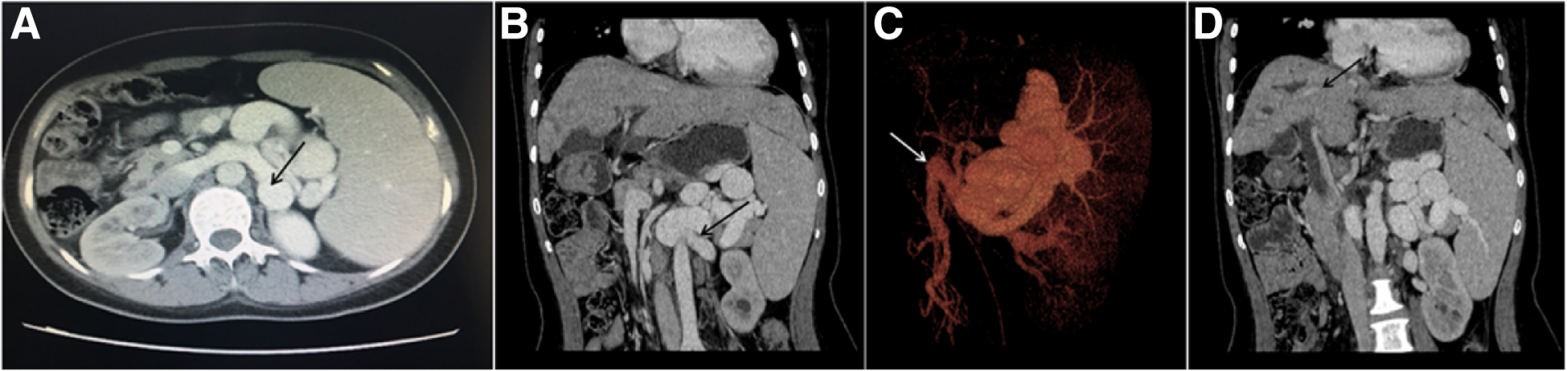
Axial, coronal and three-dimensional CT images show type II Abernethy malformation in the patient. (**a** and **b**) A side-to-side shunt between the splenic vein and the left renal vein is visible in axial and coronal CT images (black arrow). **c** 3D vessel reconstruction shows a normal main portal vein formed from the splenic and superior mesenteric veins (arrow). **d** The portal vein appears slender in the coronal CT image (arrow)

**Fig. 2:**
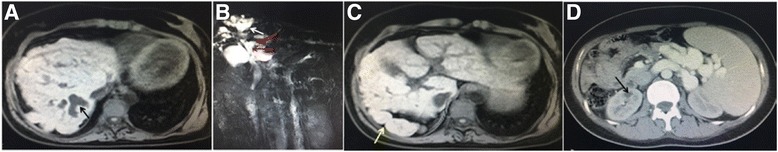
The patient’s radiological imaging findings are consistent with Caroli’s syndrome. **a** Cross-sectional MRI images show intrahepatic bile duct dilatation (arrow). **b** MRCP shows intrahepatic bile duct dilation (white arrow) along with both common hepatic duct (upper red arrow) and common bile duct (lower red arrow) dilations. **c** Cross-sectional MRI images show hepatic fibrosis (arrow). **d** The CT image shows kidney cysts (arrow)

A 23-year-old woman was admitted to our hospital with a history of longer than 1 year of fatigue, a dim complexion, and mild anorexia. She had no history of hypertension, heart disease, blood transfusions or surgery. A three-generation pedigree did not reveal a history of hepatitis. She had no jaundice. The results of liver function tests were as follows: alanine aminotransferase 22 U/L, aspartate aminotransferase 25 U/L, gamma glutamyl transferase 36 U/L, alkaline phosphatase 66 U/L, albumin 35 U/L, total bilirubin 24.8 μmol/L and direct bilirubin 10.9 μmol/L. She had a reduced level of cholinesterase (4343 U/L). A viral hepatitis panel was negative. Her serum copper level was also in the normal range. Autoimmune antibody and immunoglobulin screens were negative. Radiological imaging was performed. CT showed a side-to-side portocaval shunt between the splenic and left renal veins (Fig. 1a and b, arrow). Three-dimensional (3D) vessel reconstruction revealed a normal orientation of the splenic and superior mesenteric veins(Fig. 1c, arrow). The main portal vein was present but slender (Fig. 1d, arrow). The morphology observed was consistent with type II Abernethy malformation. MRI was undertaken to further characterise the disorders in the patient, and cross-sectional MRI images revealed intrahepatic bile duct dilation in the right lobe of the liver (Fig. 2a, arrow) and hepatic fibrosis (Fig. 2c, arrow). CT scans also showed kidney cysts (Fig. 2d, arrow). These findings are consistent with a diagnosis of Caroli’s syndrome. Magnetic resonance cholangiopancreatography (MRCP) also confirmed ectasia of the intrahepatic bile duct (Fig. 2b, white arrow) and common hepatic/bile duct dilation (Fig. 2b, red arrow).

**Fig. 3:**
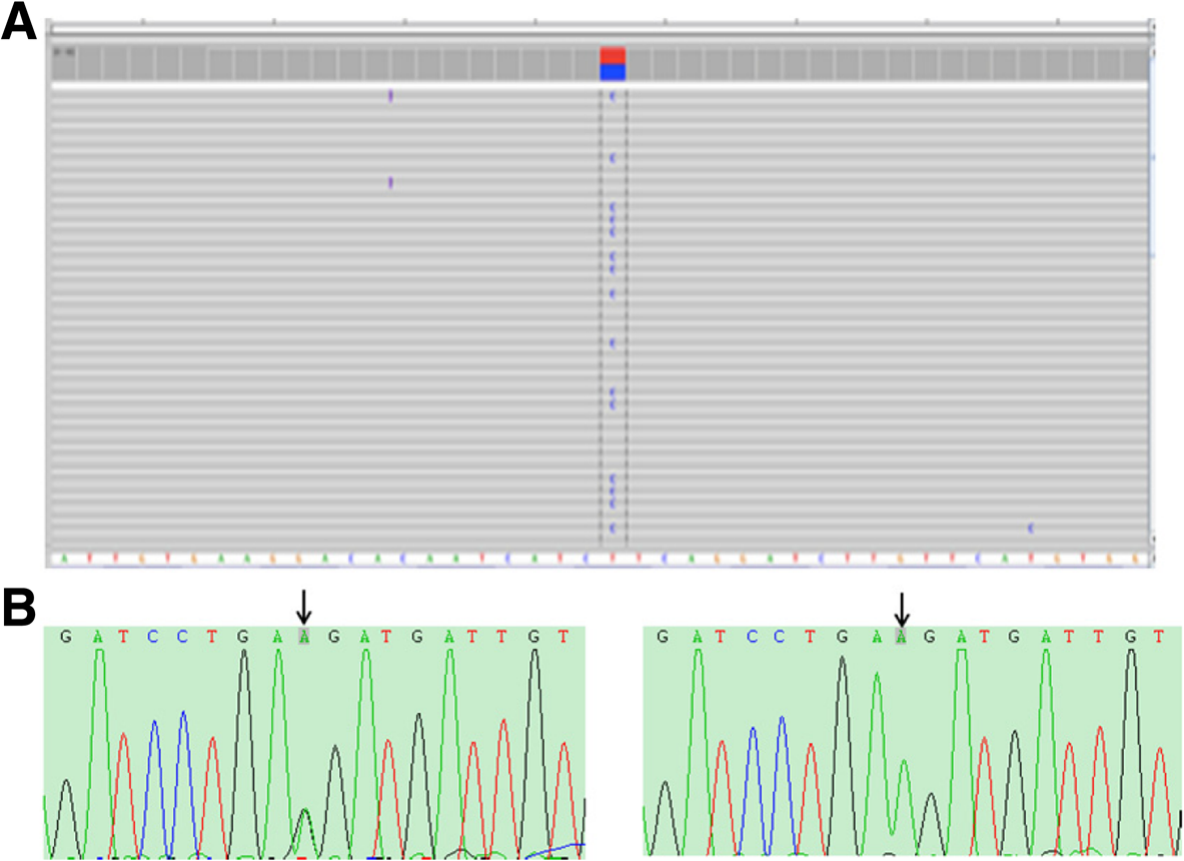
Genetic characterisation of Caroli’s syndrome was performed via *PKHD1* mutational analysis. **a** Results from next-generation sequencing of the patient’s genome. A heterozygous mutation (A > G, frequency: 61.45%) was detected in exon 20 (c.1877) of the *PKHD1* gene. **b** Parental mutation analysis in *PKHD1* exon 20. The same heterozygous missense mutation (c.1877A > G, p.Lys626Arg) was detected in the father (arrow, left), while the mother carried the wild-type genotype (arrow, right)

Mutational analysis of the *PKHD1* gene was performed to identify any genetic anomalies. Genomic DNA was extracted from the peripheral blood leukocytes of both the patient and her parents. Next-generation (NextGen) sequencing was undertaken. A sequencing library was constructed using an Ion AmpliSeq™ Library Kit 2.0 from Life Technologies. Sequencing was carried out with an Ion Torrent PGM™ 200 Sequencing Kit in an Ion Torrent PGM™ System from Life Technologies, and data analysis was performed using BWA-0.7.12 software with the mem comparison strategy following the Genome Analysis Toolkit best practices developed by the Broad Institute. A heterozygous missense mutation in exon 20 (c.1877A > G, p.Lys626Arg) of *PKHD1* was identified in the patient (Fig. 3a). We further performed a parental analysis in the targeted mutation site via PCR-based exon amplification and direct bidirectional sequencing of both strands of *PKHD1* using an ABI 3730XL DNA Analyzer. The same heterozygous missense mutation was identified in the father, and the wild-type genotype was identified in the mother (Fig. 3b).

**Fig. 4:**
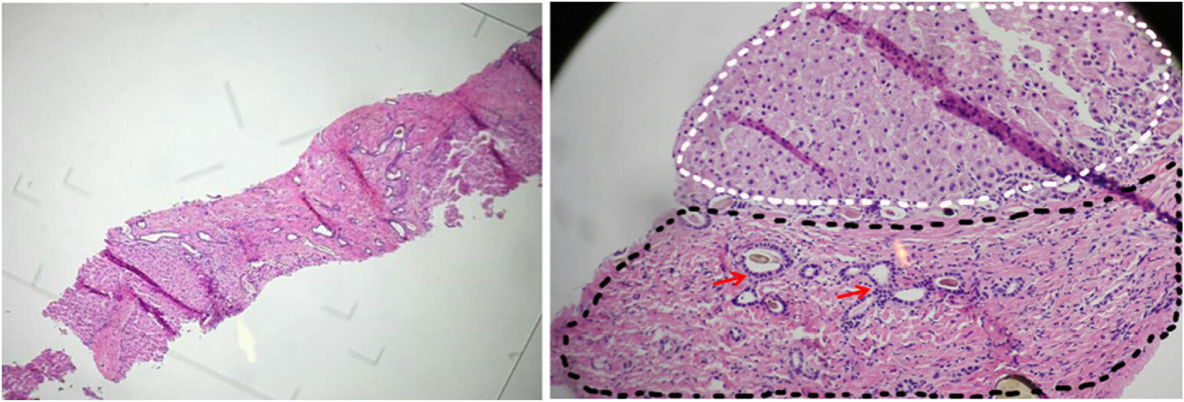
Liver biopsy confirmed the features of Caroli’s syndrome in the patient. Liver biopsy showed thick, bridging fibrosis with bile ductular proliferation and dilation (the black dots indicate the portal area; the red arrow shows dilated bile ducts), and fibrous bands traversed the hepatic parenchyma, forming nodules (non-regenerative; indicated by the white dots). Left, lower magnification; right, higher magnification

A liver biopsy was performed, and it revealed thick fibrous bands containing bile ductular proliferation along with duct dilation (Fig. 4). The lobular parenchyma was irregularly shaped and appeared to be separated and surrounded by fibrosis bands, with visible nodules (Fig. 4). Based on the radiological imaging, genetic testing and liver biopsy, a diagnosis of type II Abernethy malformation associated with Caroli’s syndrome was made.

## Discussion

Abernethy malformation is a rare congenital vascular malformation involving the diversion of portal blood away from the liver. Patients with Abernethy malformation exhibit variable clinical presentations, ranging from having no symptoms to having nonspecific symptoms such as acute hepatic decompensation or cirrhosis [8]. The early recognition of Abernethy malformation is important as the limited portal blood supply increases the risk of hepatic neoplasms, such as hepatocellular carcinoma and hepatoblastoma, which are more frequently associated with type I Abernethy malformation [14, 15]. It is important to not only detect portal vein malformations but also identify other associated anomalies. A diagnosis of Abernethy malformation can now be made via noninvasive imaging technologies, including ultrasound, CT, and MRI. The 3D reconstruction of blood vessels is very useful for demonstrating extrahepatic portocaval shunts in patients who are suspected of having Abernethy malformation [16]. Kong et al. provided an informative case in which a 28-year-old woman was diagnosed with type II Abernethy malformation and multiple aneurysms via imaging [17]. Abernethy malformation requires high-quality imaging methods to identify details in the systemic and portal circulation and confirm a diagnosis. However, imaging technologies such as CT and MRI are absent from many health centres. We subjected the patient in this case report to meticulous imaging diagnostics in our hospital. In this patient, 3D-CT reconstruction showed a normal confluence of the splenic vein and superior mesenteric vein with a side-to-side portosystemic shunt, which is the characteristic morphology of a type II Abernethy malformation. In addition, cross-sectional MRI images revealed intrahepatic bile duct dilation and hepatic fibrosis in the patient, which are classic features of Caroli’s syndrome. Furthermore, MRCP showed clear intrahepatic bile duct dilation and enlarged common hepatic/bile ducts. The main portal vein was slender, but its branches were not clearly visible in our images.

Genetic evaluation and liver biopsy are the gold standards for diagnosing Caroli’s syndrome, which is closely associated with congenital hepatic fibrosis, and up to 60% of reported cases have renal involvement [13, 18]. A close link between Caroli’s syndrome and *PKHD1* gene mutations has been reported in the literature [19, 20]. *PKHD1* encodes fibrocystin/polycystin proteins that are mainly expressed in the renal and biliary epithelia, and are predicated to have a role in the maintenance of 3D tubular architecture [21]. *PKHD1* is one of the longest human genes, extending over 469 kb, with the longest transcript encompassing 67 exons [22]. Given that mutations are spread throughout the *PKHD1* gene and not clustered in a single region, screening for *PKHD1* mutations is challenging, making it necessary to use NextGen sequencing [23]. This makes it possible to evaluate several genes of interest (*HNF1B*, *PKD1* and *PKD2*) in a single test. In our case, we applied NextGen sequencing to detect *PKHD1* mutations and used Sanger sequencing of PCR fragments in a parental *PKHD1* targeted-region mutational analysis. We detected one missense mutation (c.1877A > G, p.Lys626Arg) in the patient and her father. This missense mutation has been recorded in the public ARPKD (autosomal recessive polycystic kidney disease)/*PKHD1* database. Although this mutation is defined as a polymorphism (*p*-value: 1.0), its pathogenicity is currently classified as undetermined in the database due to an insufficient number of reported related cases. Jang et al. reported the only other case, which involved a fetus at 22 weeks of gestation with enlarged kidneys identified via ultrasonographic examination, in which the same heterozygous missense mutation (Lys626Arg) has been verified via *PKHD1* mutation analysis in a patient and their father [24]. We provide a second case in a patient from Asia with liver and renal disease who carried the same paternally inherited missense *PKHD1* mutation.

Liver biopsy is useful not only to reveal fibrosis associated Caroli’s syndrome but also to give a clear view of portal vein branches [25]. In this case, liver biopsy confirmed fibrosis that was associated with bile duct dilation and ductular proliferation, which are characteristics of Caroli’s syndrome. The intrahepatic section of the portal vein was not clearly visible.

The underlying pathogenic mechanism of the combination of the two types of malformations in the present case remains unclear. Patients with type II Abernethy malformation have a hypoplastic portal vein and hypertrophic hepatic arteries. The increase in arterial blood flow compensates for the loss of portal blood flow but leads to blood vessel remodelling [26]. It has been suggested that blood vessel remodelling may provide an environment for the development of neoplastic tumours in patients [26, 27]. In the patient in the present case, hypertrophy of the hepatic arteries was observed. *PKHD1* mutations mainly lead to ductal plate malformation of the liver and renal collecting duct dilation [21]. Whether the *PKHD1* mutation identified in the present case is directly related to Abernethy malformation needs further investigation. If it does, it is likely that blood vessel remodelling occurring alongside *PKHD1* mutation contributes to the combination of the two types of malformation observed in this case.

The treatment of Abernethy malformation depends on the type of shunt present and the associated conditions. For patients with type I Abernethy malformation, liver transplantation is the only effective solution for those who develop severe hepatic encephalopathy or malignant tumours [28]. For patients with type II Abernethy malformation, shunt occlusion may be performed in cases with serious symptoms, such as hepatic encephalopathy [29]. For the patient in the present case, our recommendations were routine clinical assessments, with regular liver function testing and intrahepatic portal vein blood flow evaluations. The patient was informed about the future possibility of shunt occlusion.

## Conclusions

In summary, we report a case of type II Abernethy malformation associated with Caroli’s syndrome. Similar cases have not been reported previously. This case highlights the value of radiological imaging, pathological examination, and genetic evaluation in the diagnosis of rare diseases.

## Abbreviations

*ARPKD*: Autosomal recessive polycystic kidney disease
*CT*: Computed tomography
*IVC*: Inferior vena cava
*MRCP*: Magnetic resonance cholangiopancreatography
*MRI*: Magnetic resonance imaging
*PKHD1*: Polycystic kidney and hepatic disease 1

## References

[1] Abstracts of the 26th Annual Conference of APASL (2017). February 15-19, 2017, shanghai, China. Hepatol Int.

[2] Abernethy J (1793). J banks. Account of two instances of uncommon formation in the viscera of the human body. Phil Trans R Soc Lond.

[3] Hao Y, Hong X, Zhao X (2015). Congenital absence of the portal vein associated with focal nodular hyperplasia of the liver and congenital heart disease (Abernethy malformation): a case report and literature review. Oncol Lett.

[4] Morgan G, Superina R (1994). Congenital absence of the portal vein: two cases and a proposed classification system for portasystemic vascular anomalies. J Pediatr Surg.

[5] Murray CP, Yoo SJ, Babyn PS (2003). Congenital extrahepatic portosystemic shunts. Pediatr Radiol.

[6] Mistinova J, Valacsai F, Varga I (2010). Congenital absence of the portal vein--case report and a review of literature. Clin Anat.

[7] Singhal M, Lal A, Thapa BR, Prakash M, Shanbhogue KP, Khandelwal N (2008). Congenital atresia of portal vein with portocaval shunt associated with cardiac defects, skeletal deformities, and skin lesions in a boy. J Pediatr Surg.

[8] Witters P, Maleux G, George C, Delcroix M, Hoffman I, Gewillig M, Verslype C, Monbaliu D, Aerts R, Pirenn J (2008). Congenital veno-venous malformations of the liver: widely variable clinical presentations. J Gastroenterol Hepatol.

[9] Kobayashi N, Niwa T, Kirikoshi H, Fujita K, Yoneda M, Saito S, Nakajima A (2010). Clinical classification of congenital extrahepatic portosystemic shunts. Hepatol Res.

[10] Desmet VJ (1998). Ludwig symposium on biliary disorders--part I. Pathogenesis of ductal plate abnormalities. Mayo Clin Proc.

[11] Veigel MC, Prescott-Focht J, Rodriguez MG, Zinati R, Shao L, Moore CA, Lowe LH (2009). Fibropolycystic liver disease in children. Pediatr Radiol.

[12] Adeva M, El-Youssef M, Rossetti S, Kamath PS, Kubly V, Consugar MB, Milliner DM, King BF, Torres VE, Harris PC (2006). Clinical and molecular characterization defines a broadened spectrum of autosomal recessive polycystic kidney disease (ARPKD). Medicine (Baltimore).

[13] Kerkar N, Norton K, Suchy FJ (2006). The hepatic fibrocystic diseases. Clin Liver Dis.

[14] Howard ER, Davenport M (1997). Congenital extrahepatic portocaval shunts--the Abernethy malformation. J Pediatr Surg.

[15] Kumar A, Kumar J, Aggarwal R, Srivastava S (2008). Abernethy malformation with portal vein aneurysm. Diagn Interv Radiol.

[16] Konstas AA, Digumarthy SR, Avery LL, Wallace KL, Lisovsky M, Misdraji J, Hahn PF (2011). Congenital portosystemic shunts: imaging findings and clinical presentations in 11 patients. Eur J Radiol.

[17] Kong Y, Zhang H, Liu C, Wu D, He X, Xiao M, Zhao G, Zhang H (2013). Abernethy malformation with multiple aneurysms: incidentally found in an adult woman with Caroli's disease. Ann Hepatol.

[18] Yonem O, Bayraktar Y (2007). Clinical characteristics of Caroli's syndrome. World J Gastroenterol.

[19] Guay-Woodford LM, Desmond RA (2003). Autosomal recessive polycystic kidney disease: the clinical experience in North America. Pediatrics.

[20] Gunay-Aygun M, Avner ED, Bacallao RL, Choyke PL, Flynn JT, Germino GG, Guay-Woodford L, Harris P, Heller T, Ingelfinger J (2006). Autosomal recessive polycystic kidney disease and congenital hepatic fibrosis: summary statement of a first National Institutes of Health/Office of Rare Diseases conference. J Pediatr.

[21] Turkbey B, Ocak I, Daryanani K, Font-Montgomery E, Lukose L, Bryant J, Tuchman M, Mohan P, Heller T, WA G, Choyke PL, Gunay-Aygun M (2009). Autosomal recessive polycystic kidney disease and congenital hepatic fibrosis (ARPKD/CHF). Pediatr Radiol.

[22] Onuchic LF, Furu L, Nagasawa Y, Hou X, Eggermann T, Ren Z, Bergmann C, Senderek J, Esquivel E, Zeltner R (2002). PKHD1, the polycystic kidney and hepatic disease 1 gene, encodes a novel large protein containing multiple immunoglobulin-like plexin-transcription-factor domains and parallel beta-helix 1 repeats. Am J Hum Genet.

[23] Tavira B, Gomez J, Malaga S, Santos F, Fernandez-Aracama J, Alonso B, Iglesias S, Benavides A, Hernando I, Plasencia A, Alvarez V, Coto E (2015). A labor and cost effective next generation sequencing of PKHD1 in autosomal recessive polycystic kidney disease patients. Gene.

[24] Jang DG, Chae H, Shin JC, Park IY, Kim M, Kim Y (2011). Prenatal diagnosis of autosomal recessive polycystic kidney disease by molecular genetic analysis. J Obstet Gynaecol Res.

[25] Collard B, Maleux G, Heye S, Cool M, Bielen D, George C, Roskams T, Van Steenbergen W (2006). Value of carbon dioxide wedged venography and transvenous liver biopsy in the definitive diagnosis of Abernethy malformation. Abdom Imaging.

[26] Kondo F, Nagao T, Sato T, Tomizawa M, Kondo Y, Matsuzaki O, Wada K, Wakatsuki S, Nagao K, Tsubouchi H, Kobayashi H, Yasumi K, Tsukayama C, Suzuki M (1998). Etiological analysis of focal nodular hyperplasia of the liver, with emphasis on similar abnormal vasculatures to nodular regenerative hyperplasia and idiopathic portal hypertension. Pathol Res Pract.

[27] Lisovsky M, Konstas AA, Misdraji J (2011). Congenital extrahepatic portosystemic shunts (Abernethy malformation): a histopathologic evaluation. Am J Surg Pathol.

[28] Franchi-Abella S, Branchereau S, Lambert V, Fabre M, Steimberg C, Losay J, Riou JY, Pariente D, Gauthier F, Jacquemin E, Bernard O (2010). Complications of congenital portosystemic shunts in children: therapeutic options and outcomes. J Pediatr Gastroenterol Nutr.

[29] Lautz TB, Tantemsapya N, Rowell E, Superina RA (2011). Management and classification of type II congenital portosystemic shunts. J Pediatr Surg.

